# 471. In-Depth Characterization of SARS-CoV-2 Variants Causing Breakthrough COVID-19 Among Hospitalized Immunocompromised (IC) Patients with or without Prior Exposure to Tixagevimab-Cilgavimab (T/C) Pre-Exposure Prophylaxis (PrEP)

**DOI:** 10.1093/ofid/ofad500.541

**Published:** 2023-11-27

**Authors:** Ghady Haidar, Jana L Jacobs, Erin Salese, Justin Ludwig, Amy Heaps, Urvi Parikh, Rahil Sethi, Lori Caruso, Haley Camacho, Tina Chinakarn, Stacey Edick, Dawn Fischer, Kailey Hughes Kramer, Amy Lukanski, Kiersten Marks, Naomi Saenz-Morales, Sara Sierra, Cátia Ferreira, Lisa Glasser, Kathleen Heil, Carla Talarico, Sylvia Taylor, Erin K McCreary, John W Mellors

**Affiliations:** University of Pittsburgh School of Medicine, Pittsburg, PA; Department of Medicine, University of Pittsburgh School of Medicine, Pittsburgh, PA, USA, Pittsburgh, Pennsylvania; Division of Infectious Diseases, University of Pittsburgh, Pittsburgh, PA, USA, Pittsburgh, Pennsylvania; Office of Quality and Clinical Research Innovation, University of Pittsburgh Medical Center, Pittsburgh, PA, USA, Pittsburgh, Pennsylvania; Division of Infectious Diseases, Department of Medicine, University of Pittsburgh School of Medicine, Pittsburgh, PA, USA, Pittsburgh, Pennsylvania; University of Pittsburgh, Pittsburgh, Pennsylvania; Department of Biomedical Informatics, University of Pittsburgh, Pittsburgh, PA, USA, Pittsburgh, Pennsylvania; Division of Infectious Diseases, Department of Medicine, University of Pittsburgh School of Medicine, Pittsburgh, PA, USA, Pittsburgh, Pennsylvania; Office of Quality and Clinical Research Innovation, University of Pittsburgh Medical Center, Pittsburgh, PA, USA, Pittsburgh, Pennsylvania; Office of Quality and Clinical Research Innovation, University of Pittsburgh Medical Center, Pittsburgh, PA, USA, Pittsburgh, Pennsylvania; Division of Infectious Diseases, Department of Medicine, University of Pittsburgh School of Medicine, Pittsburgh, PA, USA, Pittsburgh, Pennsylvania; Office of Quality and Clinical Research Innovation, University of Pittsburgh Medical Center, Pittsburgh, PA, USA, Pittsburgh, Pennsylvania; Division of Infectious Diseases, Department of Medicine, University of Pittsburgh School of Medicine, Pittsburgh, Pennsylvania; Office of Quality and Clinical Research Innovation, University of Pittsburgh Medical Center, Pittsburgh, PA, USA, Pittsburgh, Pennsylvania; Office of Quality and Clinical Research Innovation, University of Pittsburgh Medical Center, Pittsburgh, PA, USA, Pittsburgh, Pennsylvania; University of Pittsburgh Medical Center, Pittsburgh, PA, USA, Pittsburgh, Pennsylvania; Office of Quality and Clinical Research Innovation, University of Pittsburgh Medical Center, Pittsburgh, PA, USA, Pittsburgh, Pennsylvania; Vaccines and Immune Therapies, BioPharmaceuticals Medical, AstraZeneca, Wilmington, DE, USA, Wilmington, Delaware; Vaccines and Immune Therapies, BioPharmaceuticals Medical, AstraZeneca, Wilmington, DE, USA, Wilmington, Delaware; Vaccines and Immune Therapies, BioPharmaceuticals Medical, AstraZeneca, Wilmington, DE, USA, Wilmington, Delaware; Vaccines and Immune Therapies, BioPharmaceuticals Medical, AstraZeneca, Gaithersburg, MD, USA, Gaithersburg, Maryland; Medical Evidence, Vaccines and Immune Therapies Unit, AstraZeneca, Cambridge, UK, Cambridge, England, United Kingdom; UPMC, Pittsburgh, PA; University of Pittsburgh School of Medicine, Pittsburg, PA

## Abstract

**Background:**

PrEP with T/C can prevent COVID-19 hospitalization and death in IC patients (pts) up to 6 months after injection. However, in the USA, authorization of T/C PrEP was paused in Jan 2023 due to loss of in vitro activity of T/C against dominant circulating SARS-CoV-2 variants, although loss of clinical efficacy is unclear. We investigated in vivo mechanisms of viral breakthrough in hospitalized IC pts with vs without prior T/C exposure.

**Methods:**

We analyzed remnant clinical SARS-CoV-2 PCR-positive swabs and sera from IC pts hospitalized at UPMC. SARS-CoV-2 variants and mutants were determined by whole genome sequencing and anti-RBD IgG levels by an enzyme immunoassay.

**Results:**

From Mar 28, 2022, to Mar 3, 2023, 72% (174/243) of swabs were successfully sequenced from 170 pts (**Table 1**). Median age was 67 yrs; 49% were male. IC conditions included organ transplant (23%) and hematologic cancer (32%) (**Table 2**). In IC patients with sequenced swabs, 21% received T/C (**Table 3**). Variant frequency mirrored national trends (**Table 3**). BA.5, XBB.1, and BF.7 were less common in T/C vs non-T/C pts (28.57% vs 47.54%; 25.00% vs 32.43%; 2.86% vs 6.56%). BA.2 and BQ.1 were more common in T/C vs non-T/C pts (26.32% vs 16.36%; 50.00% vs 41.25%). The R346T and K444T/R/N mutations were more common in T/C vs non-T/C pts: 54% vs 41% and 37% vs 22% (**Table 3**). Anti-RBD IgG titers from 56% pts at the time of infection were higher in T/C vs non-T/C pts (median [U/mL, IQR] 1,524,000 [463,666–2,841,800] vs 433,380 [0–2,189,800], respectively). COVID-19 mortality was numerically lower in T/C vs non-T/C pts (11% [4/35] vs 21% [28/135], respectively, *P*=0.21). Mortality differences were consistent across variant epochs (**Table 1**).

**Figure:**
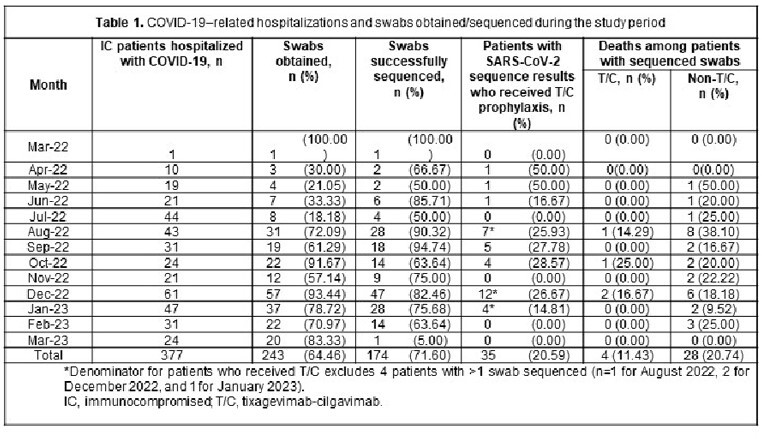

**Figure:**
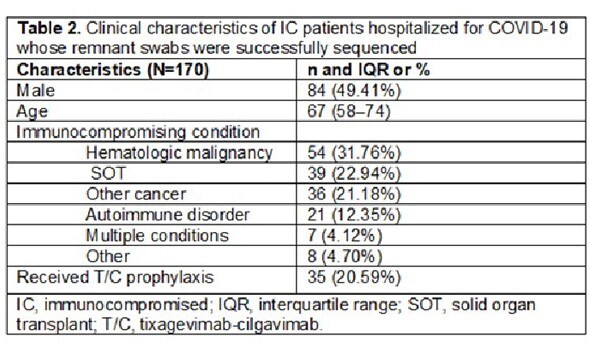

**Figure:**
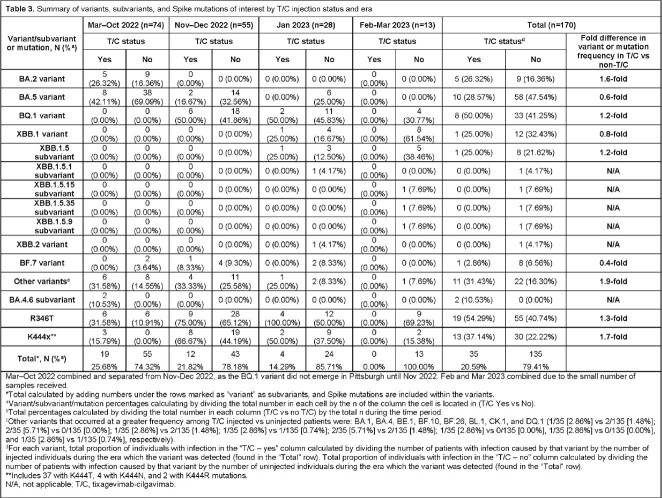

**Conclusion:**

Breakthrough COVID-19 caused by SARS-CoV-2 variants with R346T or K444T/R/N mutations is more common in IC pts who received T/C PrEP vs those who did not. Though authorization of T/C was paused due to increased prevalence of non-neutralized variants, such variants were not consistently more common in hospitalized IC pts with breakthrough COVID-19 who had received T/C. Anti-RBD IgG titers were higher and mortality was lower for T/C vs non-T/C pts. Longer follow-up is needed to further delineate the mechanisms of breakthrough infection by T/C status.

**Disclosures:**

**Ghady Haidar, MD**, Allovir: Grant/Research Support|AstraZeneca: Advisor/Consultant|AstraZeneca: Grant/Research Support|Karius: Advisor/Consultant|Karius: Grant/Research Support|NIH: Grant/Research Support **Cátia Ferreira, PhD**, AstraZeneca: Employee **Lisa Glasser, MD**, AstraZeneca: Stocks/Bonds **Kathleen Heil, RN, BSN**, AstraZeneca: Employee **Carla Talarico, PhD, MPH**, AstraZeneca: Stocks/Bonds **Sylvia Taylor, PhD, MPH, MBA**, AstraZeneca: Stocks/Bonds **Erin K. McCreary, PharmD**, Abbvie: Advisor/Consultant|Ferring: Advisor/Consultant|GSK: Honoraria|La Jolla (Entasis): Advisor/Consultant|LabSimply: Advisor/Consultant|Merck: Advisor/Consultant|Shionogi: Advisor/Consultant|Shionogi: Honoraria **John W. Mellors, MD**, AstraZeneca: Grant/Research Support|Gilead Sciences: Grant/Research Support

